# Colitis-Associated Colorectal Cancer—STAT3 Inhibition as a Preventive Strategy

**DOI:** 10.3390/ijms27188381

**Published:** 2026-09-20

**Authors:** Prema Robinson, Tan Hoang, Zara Italia, Zal Italia, Chia-Chi Chang, Ashish V. Damania, Nadim J. Ajami, Emma Rodriguez, Moses Kasembeli, Leticia Hamana Zorrilla, Luisa Maren Solis Soto, Caelyn Pham, Jacob Nguyen, Yousef Zamil, César Andrés Chávez Durán, Uddalak Bharadwaj, Rajasekaran Mahalingam, David J. Tweardy

**Affiliations:** 1 Departments of Infectious Diseases, Infection Control & Employee Health, Division of Internal Medicine, University of Texas MD Anderson Cancer Center, 1515 Holcombe Blvd., Houston, TX 77030-4009, USAmmkasembeli@mdanderson.org (M.K.); a01568680@tec.mx (C.A.C.D.);; 2 Department of Genomic Medicine, Division of Cancer Medicine, University of Texas MD Anderson Cancer Center, Houston, TX 77030-4009, USA; 3 Department of Translational Molecular Pathology, Division of Pathology of Laboratory Medicine, University of Texas MD Anderson Cancer Center, Houston, TX 77030-4009, USA; 4 Department of Biology, School of Arts and Sciences, University of St. Thomas, Houston, TX 77006, USA; 5 Department of Biology & Biochemistry, College of Natural Sciences and Mathematics, University of Houston, Houston, TX 77204, USA; 6 Department of Symptom Research, Division of Internal Medicine, University of Texas MD Anderson Cancer Center, Houston, TX 77030-4009, USA; 7 Department of Molecular & Cellular Oncology, University of Texas MD Anderson Cancer Center, Houston, TX 77030-4009, USA

**Keywords:** colorectal cancer, inflammatory bowel disease, prevention strategy, anti-cancer STAT3 inhibitor

## Abstract

Colorectal cancer (CRC) occurs with higher frequency in patients with inflammatory bowel disease (IBD) and has increased morbidity and mortality compared with CRC in patients in the general population. STAT3 has been implicated in CRC development, but strategies to target it have yet to be employed to prevent its occurrence in groups at high risk for CRC. Our group developed TTI-101, a small-molecule STAT3 inhibitor, which was effective in treating colitis in the dextran sodium sulfate (DSS) mouse model. In the current study, we examined the ability of TTI-101 to prevent CRC in the azoxymethane (AOM)-DSS mouse model of CRC. Mice received AOM followed by DSS and were treated with either TTI-101 or vehicle control for 10 weeks. While vehicle-treated AOM-DSS mice developed polyps and adenocarcinomas, TTI-101-treated AOM-DSS mice did not. Levels of activated STAT3 (pY-STAT3) were increased in both the epithelial and stromal compartments of adenocarcinomas vs. normal colon mucosa and correlated with adenocarcinoma burden. Pharmacologically relevant concentrations of TTI-101 were detected in plasma and colon; plasma levels correlated with colon levels and correlated inversely with adenocarcinoma number. Transcriptomic analyses revealed that TTI-101 normalized expression of AOM-DSS-induced CRC-associated genes in the colon, many of which are regulated by STAT3. The addition of DSS to AOM resulted in a distinct cecal microbiome diversity and composition that was muted by the addition of TTI-101. Thus, TTI-101 prevented colitis-associated CRC in the AOM-DSS model through targeting STAT3’s pro-oncogenic effects on the colon transcriptome and modulating colitis-associated dysbiosis; targeting STAT3 in patients with IBD merits consideration for CRC prevention, as well as IBD treatment.

## 1. Introduction

Colorectal cancer (CRC) is the second most common cause of cancer-related mortality in the United States [1]. In the general population, the lifetime probability of developing CRC is approximately 5%. In contrast, individuals with inflammatory bowel disease (IBD), especially ulcerative colitis (UC), exhibit a substantially increased risk estimated to be 20- to 30-fold higher than that of the general population [2]. Moreover, CRC arising in UC often presents at a more advanced stage and is associated with higher mortality [3].

In the setting of IBD, persistent inflammation within the stromal microenvironment has been proposed to drive pathological changes in the intestinal epithelium that promote malignant transformation [4]. Interleukin-6 (IL-6) is a cytokine implicated in both the pathogenesis of IBD and IBD-associated CRC. IL-6 exerts its downstream effects through activation of several signaling pathways, including signal transducer and activator of transcription (STAT) 3. Multiple clinical studies have implicated STAT3 signaling as an integral component of inflammation-associated CRC [5,6]. Elevated STAT3 protein expression has been reported in roughly 52–73% of human CRC tumors, and its expression levels correlated with tumor invasion depth, lymph node involvement, venous invasion, and CRC-specific mortality [5,6].

Experimental models support a causal role for STAT3 in CRC oncogenesis. In the azoxymethane (AOM) plus dextran sodium sulfate (DSS) model of colitis-associated CRC, mice engineered to express only the pro-inflammatory STAT3α isoform developed tumors earlier than wild-type controls [7]. Conversely, in other studies using the AOM-DSS model, mice lacking STAT3 specifically in intestinal epithelial cells exhibit significant reductions in both tumor incidence and size [8].

Our group identified TTI-101, a small-molecule inhibitor of STAT3 that directly targets the phosphotyrosyl (pY) peptide-binding pocket within the STAT3 SH2 domain [9]. By blocking this interaction, TTI-101 prevents STAT3 from binding to pY-containing motifs on activated cytokine receptors, thereby inhibiting cytokine-driven STAT3 activation [i.e., blocking its phosphorylation at tyrosine (Y) 705, pY-STAT3].

In this study, we evaluated the ability of TTI-101 to prevent tumor development in the AOM-DSS mouse model of colitis-associated CRC. TTI-101 treatment completely prevented development of polyps and adenocarcinomas without adverse effects on body weight or overall health. Levels of nuclear pY-STAT3 within both epithelial and stromal cells correlated with numbers of adenocarcinomas. Pharmacologically relevant concentrations of TTI-101 were detected in plasma and colon; plasma levels correlated with colon levels and were inversely correlated with adenocarcinoma number. Transcriptome analyses revealed that TTI-101 normalized AOM-DSS-induced gene expression changes in the colon, including genes activated by STAT3 and those associated with CRC; stool microbiome analysis revealed that TTI-101 also muted AOM-DSS-induced dysbiosis. Overall, these findings provide preclinical support for further investigating STAT3 inhibition as a potential strategy to prevent CRC in patients with IBD.

## 2. Results

### 2.1. TTI-101 Prevented Development of Polyps and Adenocarcinomas in AOM-DSS Mice

**Figure 1:**
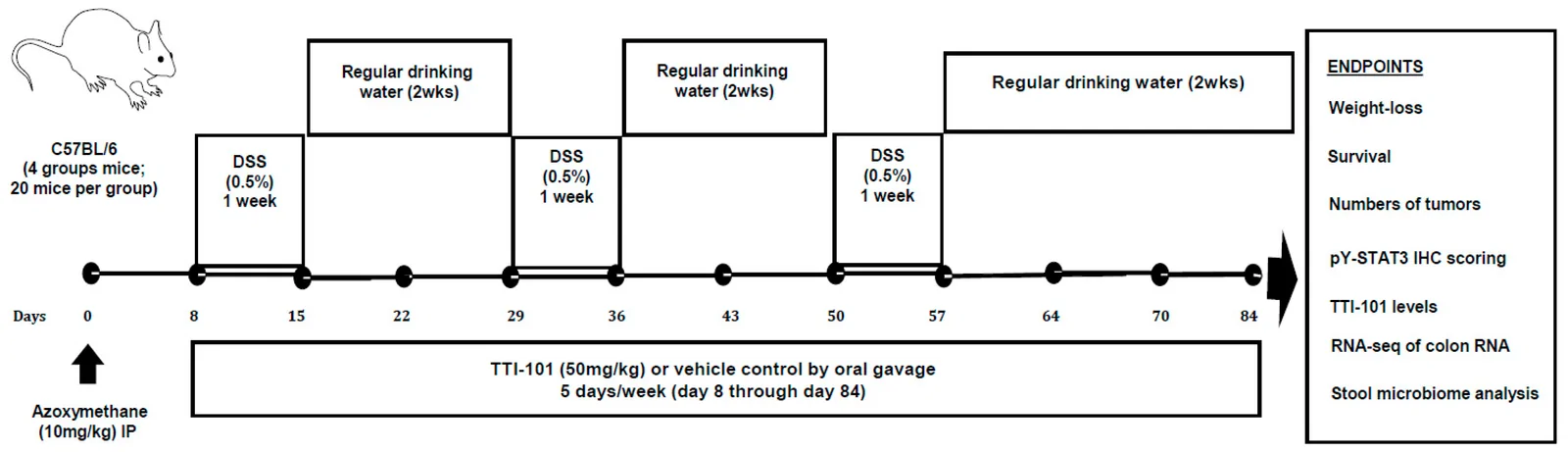
Schema of TTI-101 for concurrent prophylaxis (chemoprevention) of CRC in the AOM-DSS mouse model of colitis-induced CRC. C57BL/6 mice were entered into the AOM-DSS model of colitis-induced CRC and randomized into four groups (20 mice per group); one group of mice was given AOM alone and treated with vehicle control; one group was given AOM alone and treated with TTI-101 (50 mg/kg); one group of mice was given AOM plus DSS and treated with vehicle control; and one group of mice was given AOM plus DSS and treated with TTI-101. Mice were evaluated for weight loss and survival from day 1 to day 84 and euthanized on day 84. Their colons were examined grossly for numbers of polyps and microscopically for adenomas and adenocarcinomas; colons and plasma were examined further, as indicated.

**Figure 2:**
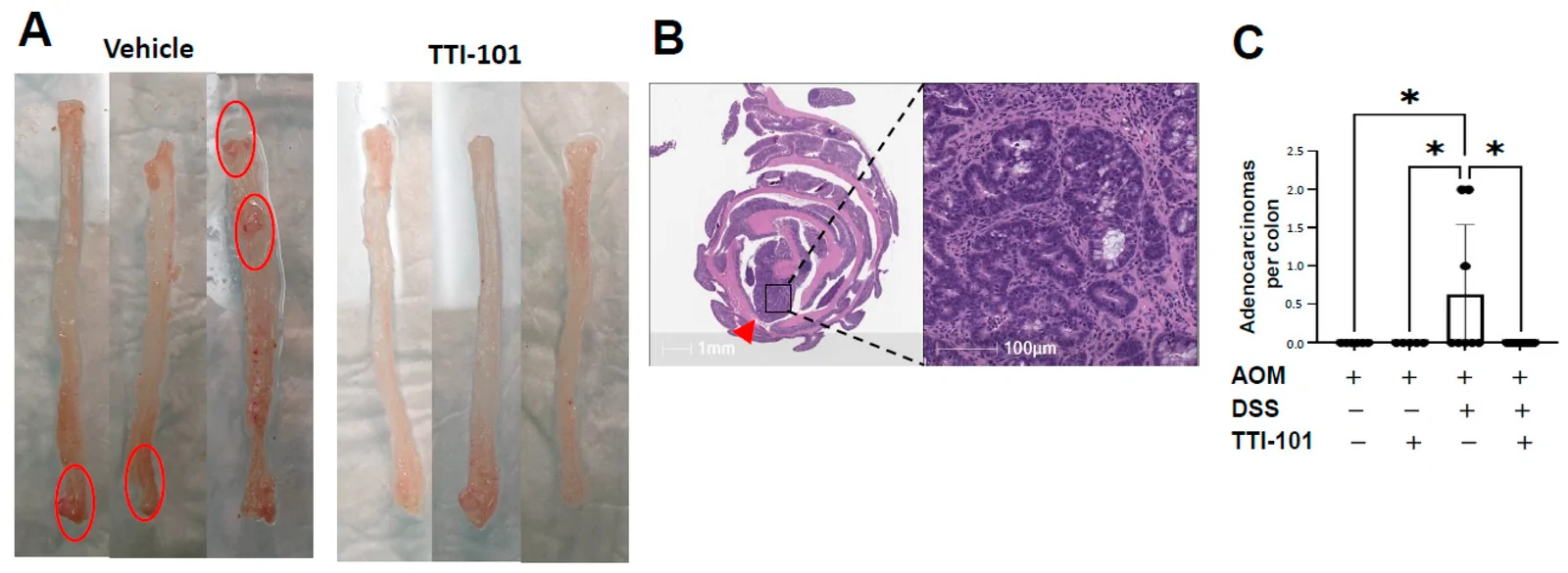
TTI-101 decreased numbers of polyps and adenocarcinomas in AOM-DSS mice. Photographs of representative colons from vehicle-treated ((**A**); (**left panel**)) and TTI-101-treated ((**A**); (**right panel**)) AOM-DSS mice (polyps circled in red). (**B**) Photomicrograph of a representative H&E section (5×; (**left panel**)) of a Swiss-rolled colon from a vehicle-treated AOM-DSS mouse showing an adenocarcinoma (red arrow); enlarged image of a portion is shown (200×; (**right panel**)). (**C**) Numbers of adenocarcinomas detected in sections of Swiss-rolled colons of mice treated as indicated (mean ± SD; *, *p* ≤ 0.05; Kruskal–Wallis test). Note: Vehicle treated mice received DSS, but no TTI-101; TTI-101 treated mice received DSS as well as TTI-101.

Eighty (80) male C57BL/6 mice (6 weeks old) were randomly assigned to four groups (*n* = 20 per group). We used male mice because female C57BL6 mice are resistant to AOM- and DSS-induced CRC [10]. Two groups received azoxymethane (AOM; 10 mg/kg, intraperitoneally, single dose), followed one week later by three cycles of dextran sodium sulfate (DSS; Figure 1). Colonic polyps were readily visible in vehicle-treated AOM-DSS mice (Figure 2A); histopathological examination of H&E-stained sections of Swiss rolled colons (Figure 2B) indicated that these polyps were adenocarcinomas (0.71 ± 0.95; mean ± SD; 3/8 mice had adenocarcinomas, one mouse had one adenocarcinoma, while the other two mice each had two adenocarcinomas; the remaining five mice did not have any adenocarcinoma; Figure 2C). In contrast, no colonic polyps were detected on gross exam in TTI-101-treated AOM-DSS mice. Importantly, histopathological examination of Swiss rolled colons revealed no adenocarcinomas (Figure 2C; *p* ≤ 0.05, Kruskal–Wallis test; *n* = 8). Similarly, no polyps or adenocarcinomas were detected in the colons of mice that received AOM alone, treated with or without TTI-101 (*n* = 5–6). There were no differences in weights, appearance, and behavior among the four mouse groups. 

### 2.2. pY-STAT3 Levels Were Increased in Nuclei of Epithelial and Stromal Cells in Adenocarcinomas, and Levels Correlated with Adenocarcinoma Numbers

**Figure 3:**
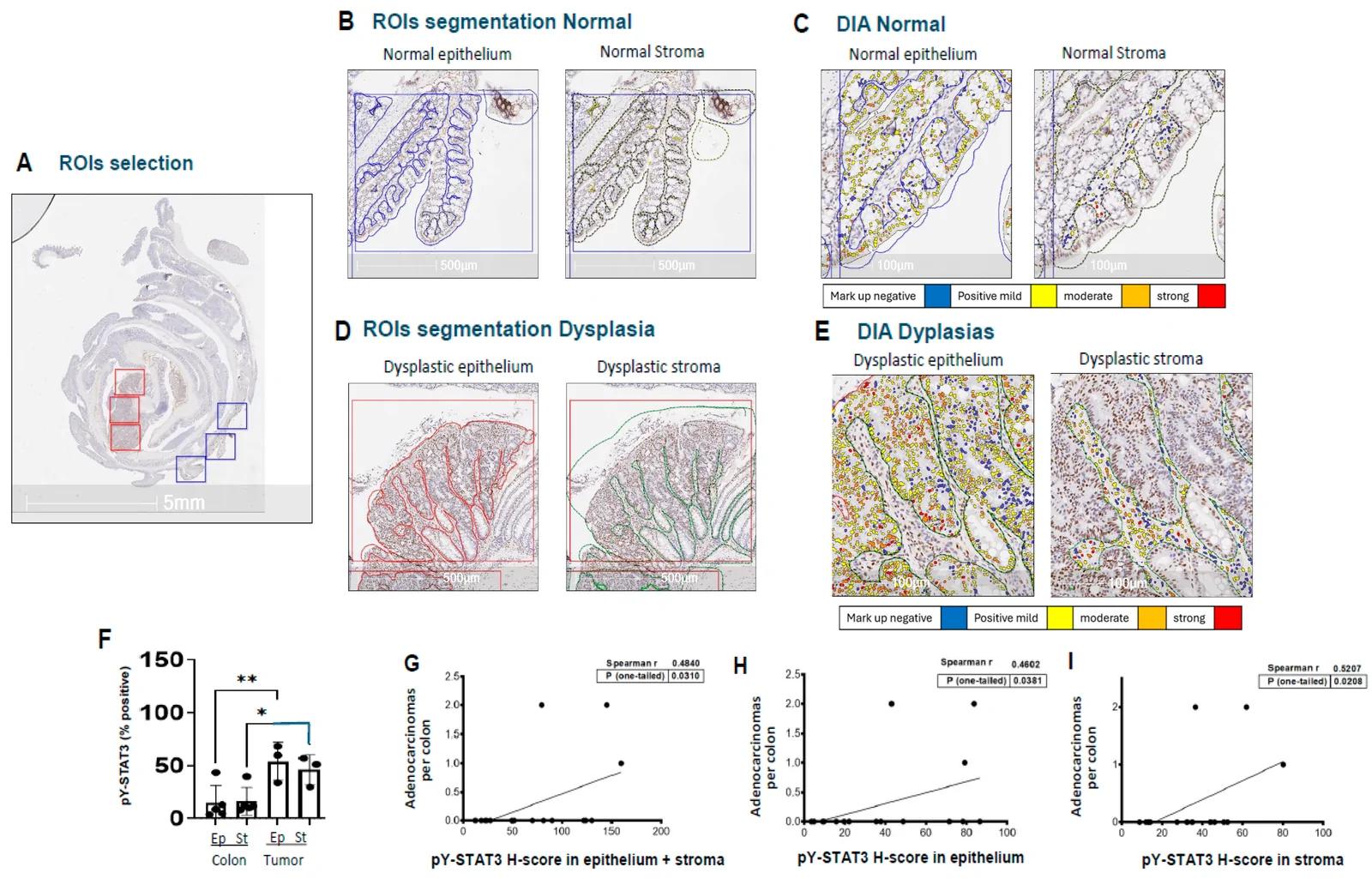
Levels of pY-STAT3 are increased in epithelial and stromal cells of adenocarcinomas (tumors) and correlated with the number of adenocarcinomas. (**A**) Regions of interest (ROIs) were selected in the normal colonic mucosa (blue) and dysplastic colonic mucosa (red; 3–5 squares each) of the colon from vehicle-treated AOM-DSS mice. Normal ROI (**B**,**C**) and dysplastic ROI (**D**,**E**) were manually segmented using annotation tools into epithelial and stromal areas of analysis (AOA; normal epithelium, blue; normal stroma, yellow; dysplastic epithelium, orange; and dysplastic stroma, Turquoise blue), Levels of nuclear pY-STAT3 in each AOA were scored at high magnification with negative nuclei marked in blue and positive cells with increasing intensities marked as follows: mild (yellow), moderate (orange) and strong (red). (**F**) pY-STAT3 levels in epithelium and stroma of normal colon and adenocarcinoma from vehicle-treated AOM-DSS mice (% positive; *^,^**, *p* < 0.05; ANOVA with pairwise comparisons using Tukey’s method). (**G**) Levels of pY-STAT3 (H-scores) within epithelium plus stroma, (**H**) epithelium alone and (**I**) stroma alone were correlated with adenocarcinoma numbers (Spearman’s and *t* tests).

Quantitative digital image analysis (DIA) of epithelial and stromal cell nuclei within adenocarcinomas and normal colonic mucosa for pY-STAT3 (Figure 3A–E) revealed increased pY-STAT3 levels in both epithelial and stromal cells (Figure 3F) of adenocarcinomas vs. normal colonic mucosa; the percentage of pY-STAT3-positive nuclei was increased by 3.6-fold in the adenocarcinoma epithelial cells (53.1 ± 17.9; *n* = 3) compared to normal mucosal epithelium (14.6 ± 16.5; *n* = 5; *p* < 0.05; ANOVA with pairwise comparisons using Tukey’s method) and was increased by 3.1-fold in the adenocarcinoma stromal cells (45.9 ± 14.4; *n* = 3; *p* < 0.05; ANOVA with pairwise comparisons using Tukey’s method) compared to normal mucosal stromal cells (16.2 ± 13.2; *n* = 5). Levels of nuclear pY-STAT3 in epithelial cells combined with stromal cells within adenocarcinomas (99.7 ± 31.6; *n* = 3; H-scores) were increased 3.2-fold compared with epithelial plus stromal cells within normal mucosa (30.7 ± 29.5; *n* = 5; *p* < 0.05); pY-STAT3 levels correlated with adenocarcinoma numbers (Figure 3G; 95%, CI, from −0.062 to 0.771). Similarly, pY-STAT3 levels in epithelial cells alone (Figure 3H; 95%, CI, from 0. 062 to 0.784) and in stromal cells alone (Figure 3I; 95%, CI, from 0.017 to 0.813) correlated with adenocarcinoma numbers (*p* ≤ 0.05; all Spearman’s and *t* tests).

### 2.3. TTI-101 Levels in Plasma and Colon

**Figure 4:**
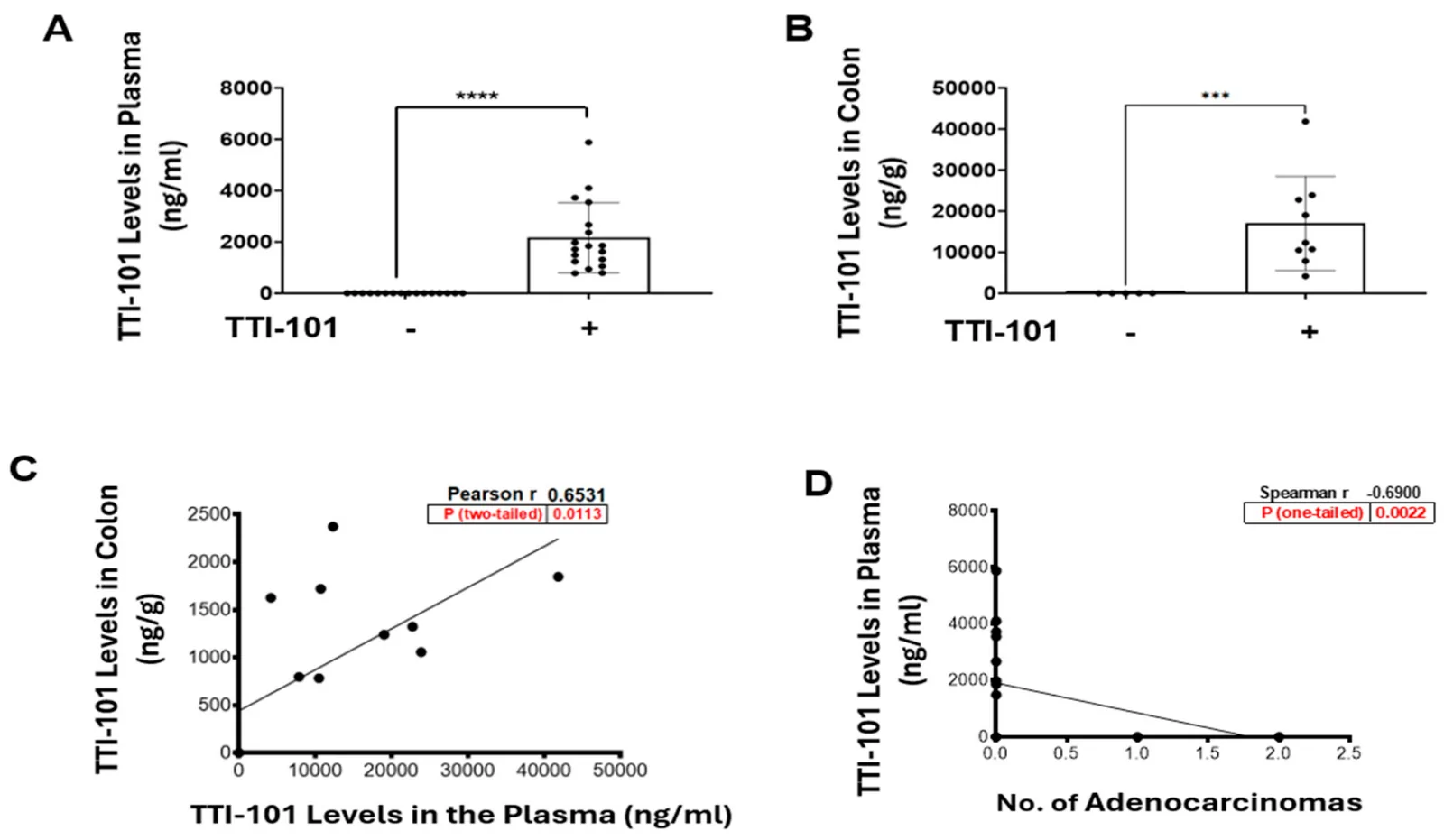
TTI-101 levels in plasma and colon. TTI-101 in (**A**) plasma and (**B**) colon of vehicle-treated and TTI-101 treated mice. (**C**) TTI-101 levels in the plasma were correlated with TTI-101 levels in the colon, and (**D**) were inversely correlated with numbers of adenocarcinoma (mean ± SD; all, *** and ****; *p* ≤ 0.05, Pearson/Spearman’s and *t* tests; *n* = 5–18). Red font in Dand E denotes significance.

TTI-101 was detected by LC-MS/MS in the t plasma (2163 ng/mL ± 1321; Figure 4A, *n* = 18) and colon (17,014 ng/g ± 10,816; Figure 4B, *n* = 9) of AOM-DSS mice treated with TTI-101 at levels that were pharmacologically relevant (IC_50_ is 717 ng/mL for inhibition of STAT3-dependent proliferation [11]. Not surprisingly, however, TTI-101 was not detected in the plasma (*n* = 16) or colon (*n* = 5) of mice treated with vehicle control (Student *t* test; *p* ≤ 0.05 for both plasma and colon). Importantly, colon levels of TTI-101 were increased ~8-fold over plasma levels, indicating drug accumulation in the target organ—a pharmacokinetic profile favorable for GI indications requiring high mucosal drug exposures. Of note, TTI-101 levels in the plasma correlated with levels in the colon [Figure 4C; 95% confidence interval (CI), 0.187–0.88; *p* ≤ 0.05, Pearson/Spearman’s and *t* tests; vehicle-treated *n* = 5, TTI-101-treated *n* = 9] and were inversely correlated with numbers of adenocarcinoma [Figure 4D; 95%, CI, from −0.88 to −0.28; *p* ≤ 0.05, Spearman’s and *t* tests; vehicle-treated *n* = 8, TTI-101-treated *n* = 8].

### 2.4. STAT3 Is a Key Driver of AOM-DSS-Induced Changes in the Colon Transcriptome; TTI-101 Reverses Most of the Changes

**Figure 5:**
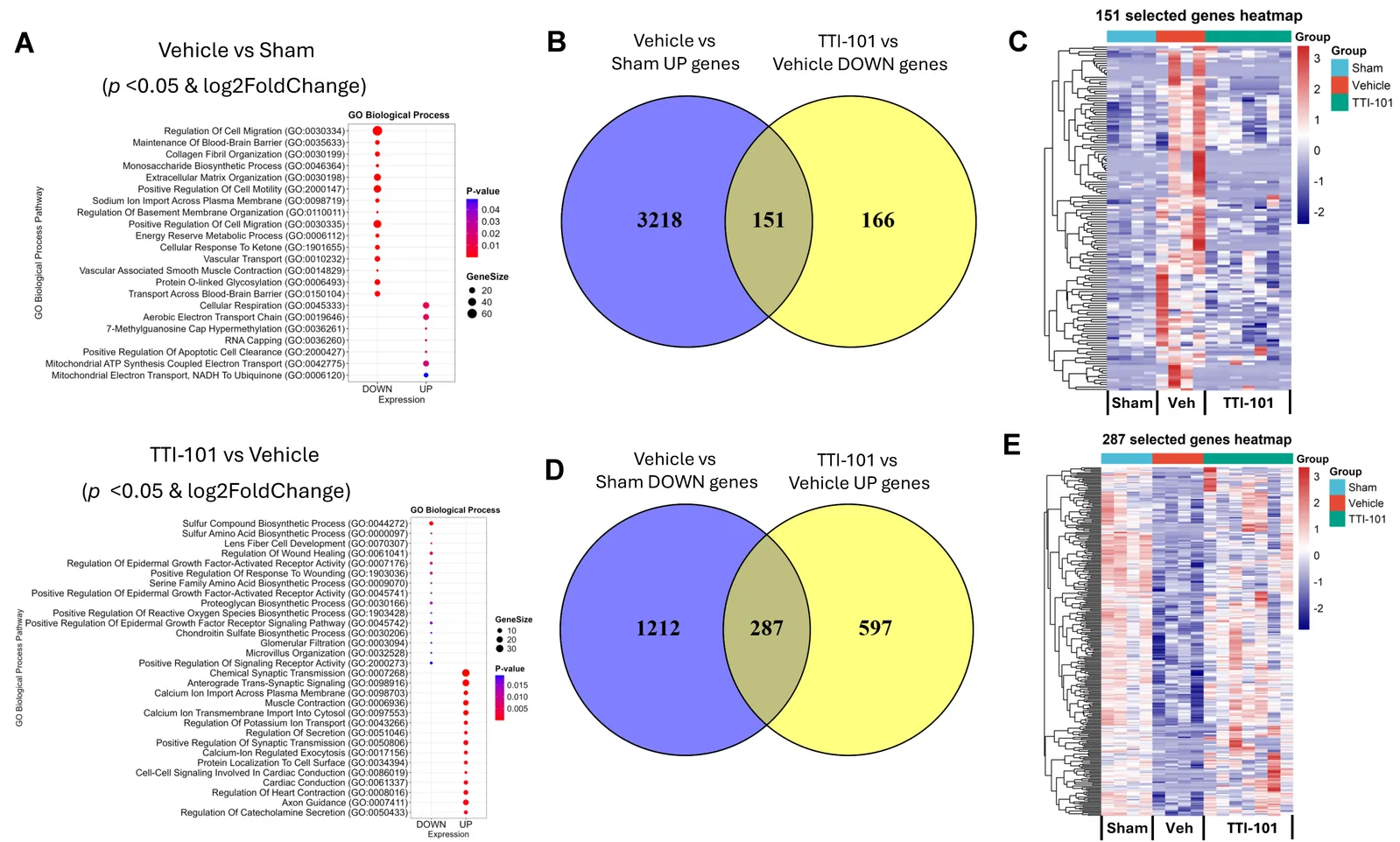
Effects of TTI-101 on colon mRNA levels. (**A**) Gene ontology (GO) of DEGs identified in Comparison 1 (Vehicle vs. Sham) and Comparison 2 (TTI-101 vs. Vehicle). (**B**,**D**) Venn diagrams depicting (**B**) the overlap of DEGs upregulated in Comparison 1 and downregulated in Comparison 2 and (**D**) the overlap of DEGs downregulated in Comparison 1 and upregulated in Comparison 2. (**C**) Heat map of the 151 DEGs whose expressions were increased in the Vehicle group and decreased by TTI-101 (UP/DOWN DEG). (**E**) Heat map of the 287 DEGs whose expressions were decreased in the Vehicle group and increased by TTI-101 (DOWN/UP DEG).

To identify candidate mRNAs that contribute to AOM-DSS-induced CRC and its prevention, we first performed two comparisons of mRNA sets: vehicle-treated AOM-DSS (Vehicle) vs. vehicle-treated AOM alone (Sham) mRNA sets (Comparison 1) and TTI-101-treated AOM-DSS (TTI-101) vs. Vehicle mRNA sets (Comparison 2) and identified differentially expressed genes (DEGs) linked by IPA to important biological processes (Figure 5A). Included in the comparisons were all coding, non-coding, and multiple complex genes that were present in all samples of both sets used in each comparison. The criteria for differential expression were *p*-values ≤ 0.05 and a log 2-fold change. We then focused on the two groups of overlapping DEGs—those increased in the Vehicle group that were decreased by TTI-101 (151 UP/DOWN DEG; Figure 5B,C) and those decreased by AOM-DSS that were increased by TTI-101 (287 DOWN/UP DEG; Figure 5D,E).

From the list of 151 UP/DOWN DEGs, we excluded those that were long non-coding RNAs (lncRNAs), pseudogenes, and newly identified but incompletely characterized genes, which left 88 well-characterized UP/DOWN DEGs. Of these 88 DEGs, STAT3 has been shown to bind to the promoters of 39 of them (44.3%; Table 1; ENCODE, ChEA database containing 15,731 genes with promoter binding by STAT3; curated from ChIP studies conducted in multiple cell/tissues systems [12,13]). Of note, 20 of these 39 DEGs are both upstream and downstream of STAT3; 13 of these 20, including PLAUR [14], ALDH3B2 [15] NME4 [16], EPHA2 [17], MAFF [18], HBEGF [19], PTP4A1 [20], SEMA7A [21], CLDN4 [22], CCL2 [23], CSF3R [24], IL1RL1 [25], RNASE1 [25], and CLEC4D [26], are linked to increased STAT3 mRNA levels, while 7 of these 20, including HIGD1A [27], SFN [28] GPRC5A [29], S100A11 [30], DSG3 [31], TRIM29 [32], and PDGFRL [33] are linked to decreased STAT3 mRNA levels. Also, of these 88 UP/DOWN DEGs, 40 DEGs are linked to CRC; 33 DEGs are linked to CRC progression and metastases, including AVPI1 [34], PLAUR [35], TRIM15 [36], ALDH3B2 [37], GPRC5A [38], S100A11 [39], IFRD1 [40], EPHA2 [41], CAPG [42], USP35 [43], RPSA-PS1 [44], DYNLT1A [45], RAP2B [46], DUSP10 [47], PTP4A1 [48], TNFRSF12A [49], SEMA7A [50], CPN1 [51], PLAT [52], RBM11 [53], RPL31-PS14 [54], CLDN4 [55], PLEK2 [56], CCL2 [57], SAA3 [58], MDFI [59], SPRR1A [60], DSG3 [61], CSF3R [62], TRIM29 [63], CLEC4D [26], PGC [64], and FMR1NB [65]. STAT3 was shown to bind to the promoters of 24 of 33 (73%) of these UP/DOWN CRC-linked DEGs (Table 1). The remaining seven UP/DOWN DEGs were shown to be protective for CRC, including HIGD1A [27], PLA2G12A [66], MAFF [67], FABP2 [68], PDGFRL [69], RNASE1 [70]; STAT3 has been shown to bind to the promoters of four of these seven (57%; Table 1).

Of the 287 DEGs that were decreased by AOM-DSS and increased by TTI-101, we excluded 49 genes that were long non-coding RNAs (lncRNAs), pseudogenes, and newly identified but incompletely characterized genes, which left 238 DOWN/UP DEGs. Of these 238 DOWN/UP DEGs, STAT3 has been shown to bind to the promoter of 123 (51.7%; ENCODE, ChEA database; curated from ChIP studies conducted in multiple cell/tissues systems [12,13]) and 82 of these 238 DOWN/UP DEGs link to CRC. Of these 82 CRC-linked DOWN/UP DEGs, 35 (43%) protect against CRC and/or are linked to better prognosis, which leaves 47 DOWN/UP DEGs (57%) that promote CRC oncogenesis (Table 2). The 35 protective DEGs include NAT1 [71], MZB1 [72], 7SK [73], CERS1 [74], KLB [75], HTR7 [76], GREM2 [77], KIF26A [78], ADD2 [79], NPY1R [80], ADH1 [81], ESRRG [82], DIRAS2 [83], SEMA3D [84], HEYL [85], KCNB1 [86], ABO [87], COL8A1 [88], THBS4 [89], GLI3 [90], PPP1R3C [91], RSPO3 [92], SYNPO2 [93], ANO1 [94], NEGR1 [95], GLI2 [96], FGFR1 [97], MMRN1 [98], SUN2 [99,100], TRPC1 [101], GDF10 [102], NRP2 [103], DCBLD2 [104,105], AKAP12 [106], FLNA [107]. STAT3 binds to the promoters of 25 of these 35 CRC-protective DOWN/UP DEGs (Table 2; [12,13]).

### 2.5. TTI-101 Treatment Is Associated with a Distinct Cecal Microbiome in AOM-DSS Mice

**Figure 6:**
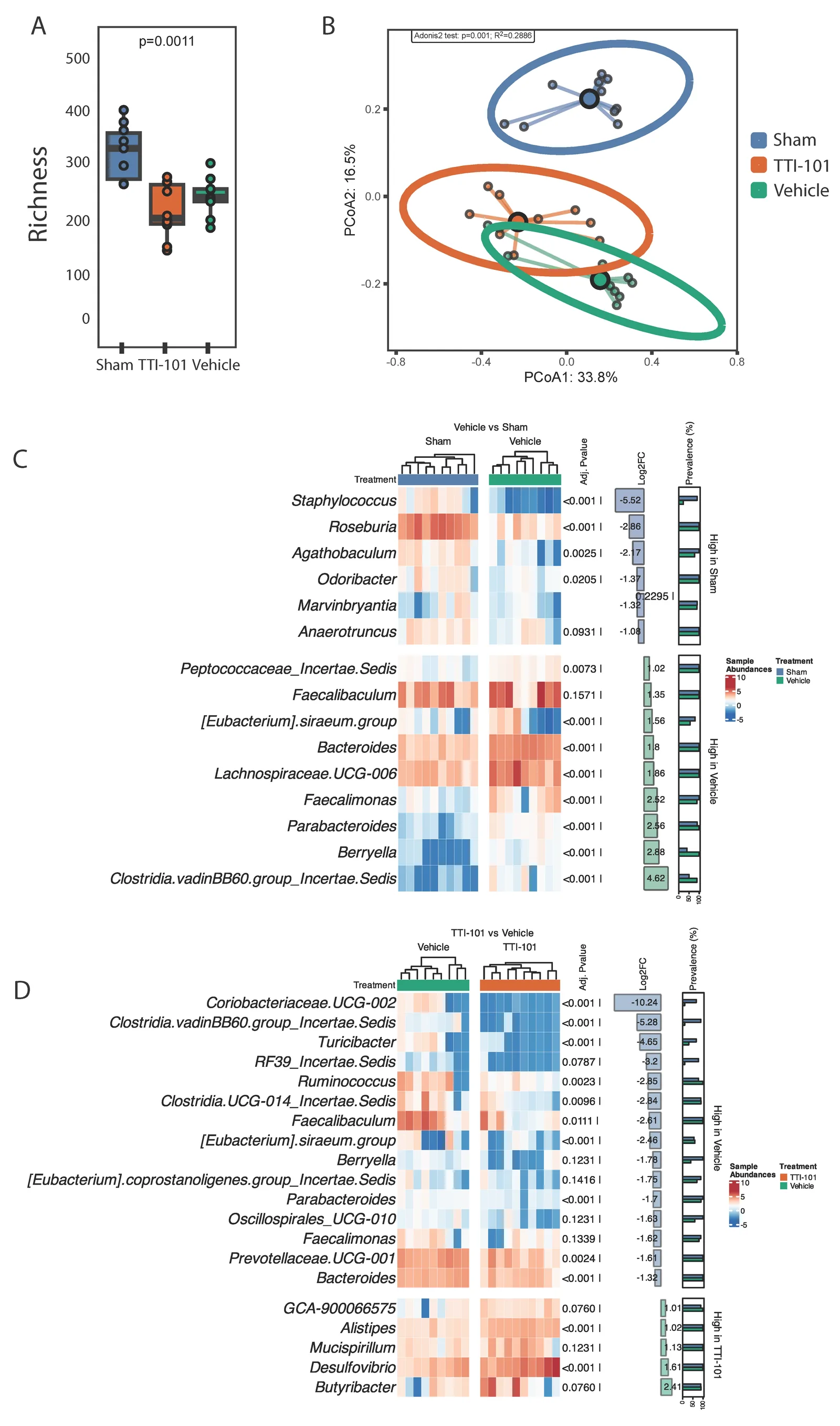
The cecal microbiome at endpoint is associated with treatment group in the AOM-DSS model. Cecal contents from Sham (AOM, *n* = 10), Vehicle (AOM-DSS, *n* = 9), and TTI-101 (AOM-DSS + TTI-101, *n* = 10) mice were collected at endpoint and profiled by 16S rRNA V4 amplicon sequencing. (**A**) ASV richness (alpha diversity) by group; *p*-values from pairwise comparisons shown. (**B**) Principal coordinates analysis (PCoA) of community composition (beta diversity); ellipses denote 95% confidence regions per group and the *p*-value reflects PERMANOVA (1000 permutations). (**C**) MaAsLin2 analysis of differentially abundant taxa between Vehicle and Sham and (**D**) between TTI-101 and Vehicle. For (**C**,**D**), adjusted *p*-values, log fold-change, and prevalence are shown alongside each taxon.

The gut microbiome from the cecum of Sham, Vehicle, and TTI-101 mouse groups (9–10 mice/group) collected at endpoint underwent 16S rRNA V4 amplicon sequencing and analysis (Figure 6); 16S rRNA V4 amplicon sequencing yielded a mean of 45,317 reads per sample (median 44,111; range 29,064–63,820), and samples were rarefied to an even depth of 29,064 reads prior to diversity analyses.

The cecal microbiome differed across the three treatment groups. Microbial richness was significantly reduced in AOM-DSS vehicle-treated mice relative to AOM vehicle-treated Sham controls (Figure 6A, *p* = 0.0022). Richness in the TTI-101 group was not significantly different from Vehicle (*p* = 0.32) and remained below Sham. Overall community structure also differed by treatment (Figure 6B). PCoA of the full community separated the three groups along the first two axes, with a significant and sizable effect (*p* = 0.001). The Sham (blue) and Vehicle (green) groups were well separated, and the TTI-101 (orange) group occupied an intermediate position between them.

Relative to Sham, Vehicle mice were depleted of a cluster of taxa that included several short-chain fatty acid (SCFA)-producing and anti-inflammatory commensals (e.g., *Roseburia*, *Agathobaculum*, *Odoribacter*, *Marvinbryantia*, *Anaerotruncus*) and were enriched for a second cluster (e.g., *Bacteroides*, *Parabacteroides*, *Faecalibaculum*, [*Eubacterium*] *siraeum* group, Lachnospiraceae UCG-006, Clostridia vadin BB60 group, *Berryella*; Figure 6C). Several taxa enriched in Vehicle (Figure 6C) were reduced in TTI-101 relative to Vehicle (Figure 6D), including Coriobacteriaceae UCG-002, Clostridia vadinBB60 group, *Turicibacter*, *Faecalibaculum*, [Eubacterium] siraeum group, *Ruminococcus*, and *Berryella*.

Taken together, these results suggest that TTI-101 may ameliorate DSS colitis in part through the microbiome via partial compositional normalization and selective reversal of specific colitogenic taxa rather than through restoration of overall diversity.

## 3. Discussion

In the current study, we examined the ability of TTI-101 to prevent CRC in the AOM-DSS mouse model of CRC. TTI-101 treatment completely prevented the development of polyps and adenocarcinomas. Levels of activated STAT3 (pY-STAT3) were increased in both the epithelial and stromal compartments of adenocarcinomas vs. normal colon mucosa of vehicle-treated mice, and pY-STAT3 levels correlated with adenocarcinoma burden. Pharmacologically relevant concentrations of TTI-101 were detected in plasma and colon; plasma levels correlated with colon levels and were inversely correlated with adenocarcinoma number. Transcriptomic analyses revealed that TTI-101 normalized AOM-DSS-induced gene expression changes in the colon, including many genes associated with CRC that are regulated by STAT3. AOM-DSS was associated with a microbiome profile characteristic of carcinogenesis; TTI-101 treatment muted this effect. Thus, TTI-101 prevented colitis-associated CRC in the AOM-DSS model, in part, through targeting STAT3’s effects on the CRC-promoting transcriptome and by modulating colitis-associated dysbiosis. These findings support further investigating STAT3 targeting as a potential strategy for CRC prevention in patients with IBD.

TTI-101 was identified by virtual-ligand docking of ~1 million compounds into the pY-peptide binding pocket of the Src-homology (SH) 2 domain of STAT3, followed by similarity screening of 3 million more compounds and hit-to-lead development that included iterative rounds of testing lead compounds for the ability to impair STAT3 function [9,108,109]; TTI-101 was determined by several other groups and us to effectively target STAT3 and to be of benefit in many mouse and rat models of STAT3-driven inflammation, fibrosis, and cancer [9,108,110,111,112,113,114,115,116,117]. A Phase I first-in-human study of TTI-101 in patients with advanced solid tumors established the recommended Phase II dose (RP2D), demonstrated a favorable safety and tolerability profile, and showed preliminary evidence of antitumor activity [118]. Several clinical studies of TTI-101 have followed the Phase I trial—three for treatment of cancer patients and one for treatment of idiopathic pulmonary fibrosis (IPF). An ongoing Phase 1b/2 REVERT liver cancer study is evaluating TTI-101 for the treatment of hepatocellular carcinoma in the first, second, and third line settings. Another ongoing study (NCT06141031) is examining TTI-101 in combination with radiotherapy in pancreatic ductal adenocarcinoma; the third study (NCT05384110) in a subset of hormone receptor-positive breast cancer patients was terminated for slow enrollment due to a change in the therapeutic landscape. The Phase II REVERT IPF study is evaluating TTI-101 for treatment of IPF. The study has completed enrollment, and preliminary results have been reported. Exploratory analyses indicated that, while no benefit was observed in forced vital capacity (FVC), patients receiving TTI-101 experienced a greater reduction in fibrosis burden, with a 9.4% decrease from baseline compared with a 2.4% reduction in the placebo group. Additionally, TTI-101 was associated with a more pronounced decrease in IL-6 levels, showing a 4.5-fold greater reduction than placebo. Given IL-6’s role as a STAT3-regulated inflammatory mediator, these findings support further investigation of TTI-101’s potential antitumor activity.

Our transcriptome results and analyses revealed that TTI-101 decreased 88 genes increased by AOM-DSS (Table 1), 33 of which are known to be associated with progressive and metastatic CRC. Published ChIP-Seq data revealed that STAT3 regulates 24 of 33 (73%) of these UP/DOWN CRC-linked DEGs. Analysis of known STAT3-regulated transcriptomic data from various tumor systems revealed an even greater number [39] of 48 (81%) of these DEGs to be STAT3-regulated by ChIP or transcriptional analysis, or both. This combined analysis revealed that 88 UP/DOWN CRC-linked DEGs increased to 59 out of 88 (67%). Downregulation by TTI-101 of these known STAT3-regulated pro-tumorigenic genes provides compelling evidence that the preventive effect of TTI-101 on polyp and adenocarcinoma in the AOM-DSS model of CRC is mediated by its targeting of STAT3.

The GO analysis revealed cell migration, extracellular matrix, and collagen fibril reorganization to be significantly increased in the diseased group, also pointing towards the role of STAT3 in the development of metastatic CRC and its successful targeting by TTI-101. The ability of TTI-101 to downregulate Plaur (Plasminogen Activator, Urokinase Receptor/uPAR), a STAT3 upregulated gene that shifts the cellular phenotype toward an invasive state through pericellular proteolysis that aids tumor metastasis and tissue remodeling in CRC [119,120,121], is notable. Expression of uPAR in tumor associated macrophages (TAM; Hazard ratio HR: 2.26 [122]) as well as in tumor cells (HR: 1.49 [122]) in the tumor core, are markers of poor prognosis in CRC; combining uPAR expression levels in TAM with soluble intact and cleaved uPAR in CRC patients’ blood leads to an even higher HR (12 or above [122]). The fact that Plaur/uPAR is both a target [123] and an upstream activator of STAT3 [14,124] suggests it is a pivotal player in the Plaur–STAT3–metastatic axis. The role of macrophages in AOM-DSS-induced CRC also merits further study as TTI-101 downregulates several other inflammatory markers that promote the growth and recruitment of TAM, including: (1) Capg (Capping Actin Protein/Macrophage-capping protein) that is induced by STAT3 and reorganizes the actin cytoskeleton, which maximizes motility and migration, promotes immunosuppression, and recruits macrophages [125,126,127]; (2) Ccl2 (Chemokine C-C Motif Ligand 2), a known STAT3-activated chemokine that recruits pro-tumorigenic M2 macrophages to the microenvironment [128,129]; (3) CSF3R, frequently upregulated in CRC, which promotes pro-tumor behavior in both tumor and immune cells and supports the polarization of macrophages toward an immunosuppressive state [130,131]; (4) serum amyloid A3 (SAA3), an acute-phase apolipoprotein that functions as a pro-inflammatory cytokine that drives cancer-associated fibroblasts (CAF) and macrophages toward a pro-tumorigenic and fibrotic microenvironment that promotes metastasis and relapse [132,133]; and (5) Il1rl1 that encodes ST2, the receptor for the alarmin cytokine IL-33, which contributes to an immunosuppressive microenvironment that dampens antitumor CD8+ T-cell activity and promotes alternative (pro-tumorigenic) macrophage polarization [132,133]. Of note, Il1rl1^−/−^ mice showed delayed AOM-DSS-induced tumor formation with fewer and smaller tumors of lower grade, compared to WT controls [132,133,134].

Gut microbiome dysbiosis is increasingly recognized as a contributor to colitis and the development of inflammation-associated CRC [135]. Our study suggests a potential mechanistic dimension to the protective effect of TTI-101 in the AOM-DSS model. TTI-101 treatment was associated with a cecal community distinct from and intermediate between the Sham and Vehicle groups, and several taxa enriched in Vehicle mice were correspondingly lower in TTI-101-treated animals. These associations are encouraging, as they are consistent with the possibility that TTI-101 modulates the gut microbiome as part of its protective mechanism. However, because samples were collected only at endpoint, we cannot determine whether these microbial differences preceded or resulted from the disease phenotype; also, 16S rRNA sequencing offers limited taxonomic resolution and no direct measure of microbial function. Nevertheless, these findings identify candidate taxa and support the microbiome as one plausible contributor to the effect of TTI-101, while awaiting confirmation from longitudinal and higher-resolution approaches.

A limitation of the present study is that TTI-101 was initiated on day 8 after AOM, before the detection of dysplasia, and was continued throughout. Our study design focused on prevention of tumor initiation and cannot directly address whether STAT3 inhibition affects progression in a colon in which dysplasia is already present, e.g., in patients with long-standing colitis under surveillance. Studies with delayed treatment initiation would be necessary to address this issue.

## 4. Material and Methods

### 4.1. TTI-101 for Concurrent Prophylaxis (Chemoprevention) in the AOM–DSS Mouse Model of Colitis-Associated CRC

Eighty (80) 6 weeks old male C57BL/6J mice (The Jackson Laboratory, Bar Harbor, ME, USA) were randomly assigned to four groups (*n* = 20 per group). Two groups received azoxymethane (AOM; 10 mg/kg, intraperitoneally, single dose, Sigma-Aldrich, St. Louis, MO, USA), followed one week later by three cycles of dextran sodium sulfate (DSS; Figure 1, MP Biochemicals, Solon, OH, USA). Each DSS cycle comprised 1 week of 0.5% DSS in the drinking water followed by 2 weeks of regular water, as previously described [8]. Beginning 8 days after AOM administration, before colorectal tumors were expected to be established, one of the AOM-DSS groups was treated daily with TTI-101 (50 mg/kg, Southwest Research Institute; SwRI, San Antonio, TX, USA) formulated in Labrasol (Gattefosse, Cedex, France; 60%) and PEG-400 (Spectrum, New Brunswick, NJ, USA; 40%) administered by oral gavage daily, while the other AOM-DSS group received vehicle control (Labrasol 60%/PEG-400 40%) on the same schedule. The remaining two groups received AOM alone (no DSS) and were subsequently treated with either TTI-101 or vehicle control as above. Clinical assessments were performed daily and included body weight, water intake, and general behavior. On study day 84, mice were humanely euthanized by exsanguination. Blood was collected with anticoagulant, separated into cellular components and plasma by centrifugation, and used to determine TTI-101 concentrations. Cecal contents were used for microbiome analyses, and colons were flushed three times with sterile 1× PBS, photographed, arranged as Swiss rolls, and fixed in neutral-buffered formalin followed by paraffin embedding (FFPE). FFPE blocks were sectioned for hematoxylin and eosin (H&E) staining and for immunohistochemistry (IHC) to detect phospho–STAT3 (pY-STAT3). The number of adenomas/adenocarcinomas was determined by histopathologic review of 5-μm H&E sections spanning the entire Swiss roll (5–9 sections per roll). Snap-frozen tissue was used for RNA extraction for RNA sequencing (RNA-seq) and lysed for TTI-101 measurements.

### 4.2. Quantification of TTI-101 Levels in Plasma and Colon

Plasma (collected as above) and lysates, prepared from sections of frozen colon, were mixed 1:1 with a stabilizer solution (in diH_2_O) containing 20 mg/mL sodium fluoride, 25 mg/mL sodium sulfite, and 25 mg/mL L-ascorbic acid, using 1× PBS to achieve the final ratio. Aliquots of plasma and tissue lysates were spiked with 5 μL of deuterated TTI-101 (TTI-101-d7) as the internal standard (IS) to a final concentration of 5 μg/mL. Calibration standards and quality-control (QC) samples were generated by adding 5 μL of the appropriate working solutions of TTI-101 and IS to 100 μL of blank normal plasma or tissue lysate. TTI-101 was extracted via one-step liquid–liquid extraction using methyl tert-butyl ether (MTBE). Following extraction, samples were dried and reconstituted in 100 μL of methanol for analysis. LC–MS/MS was performed on a QTRAP 5500 (SCIEX, Framingham, MA, USA) hybrid quadrupole–linear ion trap system equipped with a TurboIonSpray source and coupled to a SCIEX Exion LC system. Data acquisition and quantification employed Analyst software v1.6 (Redwood City, CA, USA). Chromatographic separation used a Synergi™ 4 μm Fusion-RP 80 Å C18 column (50 × 2 mm; Phenomenex, Torrance, CA, USA) maintained at 40 °C with a 3 min linear gradient at 500 μL/min. The aqueous mobile phase (A) consisted of 0.1% (*v*/*v*) formic acid and 5 mM ammonium acetate in water; the organic mobile phase (B) consisted of 0.1% formic acid and 5 mM ammonium acetate in methanol. Multiple reaction monitoring (MRM) in positive electrospray mode (ESI+) was used for TTI-101 and the IS (TTI-101-d7). The monitored transitions were *m*/*z* 472.093 > 301.1 for TTI-101 and *m*/*z* 479.200 > 301.1 for the IS. Calibration curves were constructed from the TTI-101/IS peak area ratios using linear regression with 1/X weighting over 0.03–30 μM with a regression coefficient (R^2^) of ≥0.9990. The lower limit of quantification (LLOQ) of 58.6ng/mL for plasma and tissues was determined as the lowest concentration of the calibration standards at which the precision was less than 15% and accuracy was within ±15% of the nominal concentration. Intra- and inter-day accuracy and precision were evaluated with 3 replicates at three QC concentrations on a single assay. Acceptable criteria were within 15% of precision and accuracy. LC–MS/MS reagents (methanol, water, ammonium acetate, and formic acid) were obtained from Honeywell Fluka (Morris Plains, NJ, USA), and MTBE from Sigma-Aldrich (St. Louis, MO, USA).

### 4.3. IHC Staining for pY-STAT3 and Quantitative Digital Image Analysis (DIA)

Swiss-rolled colon FFPE sections were stained for pY-STAT3, as described [136]. Briefly, sections were deparaffinized by three changes in freshly prepared xylene (10 min each), followed by graded ethanol washes (100%, 90%, 80%, 70%; 10 min each). Slides were rehydrated with 1× PBS (three rinses, 5 min each), then endogenous peroxidase activity was quenched with 3.0% hydrogen peroxide in water for 5 min at room temperature. After two PBS washes (10 min each), sections were incubated with a primary rabbit monoclonal antibody against pY-STAT3 (D3A7, XP^®^ Rabbit mAb, Cat. #9145; Cell Signaling Technology, Beverly, MA, USA). Antibody titration identified an optimal dilution of 1:200 using the automated Bond Polymer Refine Detection kit (Cat. #DS9800; Leica Biosystems, Buffalo Grove, IL, USA). This antibody has been previously validated in our hands for both IHC and immunoblotting, confirming specificity for the STAT3 pY705 epitope, as reported by the manufacturer and other groups. For digital image analysis, pY-STAT3–stained slides were scanned at 20× on an Aperio AT2 scanner (Leica Biosystems). Whole-slide images (WSIs) were analyzed with the HALO platform (Indica Labs, Albuquerque, NM, USA; v3.5.3577.108). Regions of interest (ROIs) encompassing areas of adenoma/dysplasia and normal-appearing colonic mucosa were identified by a board-certified pathologist (LHZ). For each case, 3–5 ROIs (HALO squares; 2.2 mm^2^ each) were selected and segmented into epithelial and stromal (lamina propria) areas of analysis (AOIs) using either manual annotation or a supervised, trained machine-learning classifier (random forest). Hemorrhage, necrosis, lymphoid follicles, mucosal folds, and other artifacts were excluded using the “exclusion annotation” tool. AOIs used for quantification were normal epithelium (N_E), normal stroma (N_S), dysplastic epithelium (D_E), and dysplastic stroma (D_S). Nuclear pY-STAT3 staining was quantified with the Cytonuclear v2.0.9 algorithm. Image parameters—nuclear contrast, optical density, roundness, segmentation, and staining thresholds—were iteratively tuned for optimal performance. Marked-up images were generated and reviewed by the pathologist (LHZ). Nuclear staining intensity was categorized as 0+ (blue), 1+ (yellow), 2+ (orange), and 3+ (red). The pY-STAT3 H-score was calculated as: (0 × % cells with 0+) + (1 × % cells with 1+) + (2 × % cells with 2+) + (3 × % cells with 3+), yielding a range from 0 to 300.

### 4.4. Total RNA Isolation and RNA Sequencing and Analysis

Total RNA was extracted from snap-frozen colon using the NucleoSpin RNA kit (Cat. #740,955.5; MACHEREY-NAGEL, Düren, Germany) following tissue homogenization. RNA quality and quantity were assessed on a NanoDrop One spectrophotometer (Thermo Scientific, Waltham, MA, USA).

RNA-seq was performed by Novogene (Sacramento, CA, USA). High-throughput bulk RNA sequencing of the samples yielded an average of 72.79 million reads per sample (range: 55.69 M to 110.45 M reads). All samples met the quality thresholds and were retained for downstream differential expression analysis. Reads were mapped to the mouse reference genome assembly mm39 (GRCm39) with an average alignment rate of 89.60% ± 0.87% (range: 88.06–91.70%), confirming consistent sequencing library quality across all samples.

Principal component analysis (PCA) was conducted using the pheatmap R package (RStudio Version 2026.08.2+200). Differentially expressed genes (DEGs) were identified by Novogene using Novomagic with thresholds of *p* ≤ 0.05 and |log_2_ fold-change| ≥ 1. Heatmaps were generated with pheatmap, and DEG sets were further interrogated using Ingenuity Pathway Analysis (IPA; QIAGEN Ingenuity Systems, version 1.24.1) and a compilation of 15,731 unique genes known to be STAT3-regulated, compiled at the Harmonizome website (https://maayanlab.cloud/Harmonizome/ accessed between 3–20 November 2025) by the Ma’ayan Laboratory [12,13], from two datasets: (1) 6014 target genes of STAT3 in low- or high-throughput functional studies from the CHEA (ChIP-X Enrichment Analysis (https://maayanlab.cloud/Harmonizome/gene_set/STAT3/ChEA+Transcription+Factor+Targets accessed between 3–20 November 2025) and (2) 13916 STAT3-target genes in ChIP-seq datasets from the ENCODE (encyclopedia of DNA elements) Transcription Factor Targets dataset (https://maayanlab.cloud/Harmonizome/gene_set/STAT3/ENCODE+Transcription+Factor+Targets; accessed between 3–20 November 2025) [137,138].

### 4.5. Microbiome Sequencing and Analysis

Cecal contents were weighed; DNA was extracted from 20 to 200 mg using the QIAamp Fast DNA Stool Mini Kit (Qiagen, Venlo, The Netherlands), as per the manufacturer’s protocol, with additional heating and bead-beating steps. Sequencing of the V4 hypervariable region of the 16S rRNA gene was as previously [139]. The quality and quantity of the barcoded amplicons were determined using an Agilent 4200 TapeStation system (Agilent, Santa Clara, CA, USA) and Qubit Fluorometer (Thermo Fisher Scientific, Waltham, MA, USA), following which libraries were prepared by pooling at equimolar ratios. The final libraries were sequenced with a 2 × 250 base pair paired-end protocol on the Illumina Miseq platform. Sequencing of 16S rRNA was analyzed using the DADA2 package (version 1.26) [140]. All taxonomic output files were imported into R using the phyloseq package (version 1.50.0) [141]. Differential abundance analysis was performed with MaAsLin2 (version 2) [142].

### 4.6. Statistical Analysis

Data were assessed for normality (as assessed using the Shapiro–Wilk test) and homogeneity of variance prior to hypothesis testing. For normally distributed data, Student’s *t* test, ANOVA/Tukey’s and Pearson’s correlation were applied; for non-normal distributions, the Mann–Whitney *U* test, Kruskal–Wallis and Spearman’s rank correlation were used; *p* values < 0.05 were considered statistically significant. Analyses were performed in R (v3.6.0) and GraphPad Prism (v7.0).

## 5. Conclusions

TTI-101 prevented colitis-associated CRC in the AOM-DSS model through targeting STAT3’s pro-oncogenic effects on the colon transcriptome and by modulating colitis-associated dysbiosis; these findings support further investigation of STAT3 targeting as a potential strategy for CRC prevention in patients with IBD.

## Figures and Tables

**Table 1:** DEGs (well-characterized) that were increased by DSS and decreased by TTI-101 (88 of 151 total UP/DOWN DEGs *): roles in CRC and promoter binding by STAT3.

Gene Symbol	TTI-101 vs. Veh (Log2FC)	*p*-Value	Roles in CRC	STAT3 Reg (ChIP) **^,^***
Fmr1nb	−7.6	0.0082	Progression/Metastasis	Yes
Pgc	−6.8	0.0003	Progression/Metastasis	Yes
Pin1rt1	−6.7	0.0332	NKR	
Olfr801	−6.5	0.0397	NKR	
Olfr1188	−6.4	0.0407	NKR	
Rnu12	−6.4	0.0407	NKR	Yes
Nphs2	−5.8	0.0458	NKR	
Platr14	−5.6	0.0020	NKR	
Btbd17	−5.5	0.0183	NKR	
Clec4d	−5.3	0.0212	Progression/Metastasis	Yes
Olfr471-ps1	−5.2	0.0375	NKR	
H2-M10.6	−5.0	0.0400	NKR	
Vmn1r204	−4.8	0.0121	NKR	
Rnase1	−4.7	0.0210	Protection	
Slc9a4	−4.6	0.0127	NKR	Yes
Obox3	−4.3	0.0192	NKR	
C1orf100	−4.0	0.0081	NKR	Yes
Nanos3	−3.6	0.0148	NKR	
Tnni2	−3.2	0.0106	NKR	
Capns2	−3.2	0.0371	NKR	
Rpl31-ps17	−3.1	0.0215	NKR	
Cyp4a31	−2.9	0.0398	NKR	
Il1rl1	−2.8	0.0068	NKR	
Pdgfrl	−2.8	0.0084	Protection	
Trim29	−2.8	0.0082	Progression/Metastasis	Yes
Csf3r	−2.7	0.0205	Progression/Metastasis	Yes
Tes3-ps	−2.5	0.0080	NKR	
Myl7	−2.4	0.0002	NKR	
Dsg3	−2.3	0.0027	Progression/Metastasis	Yes
Sprr1a	−2.2	0.0057	Progression/Metastasis	Yes
Mdfi	−2.2	0.0043	Progression/Metastasis	Yes
Saa3	−2.2	0.0006	Progression/Metastasis	
Ccl2	−2.1	0.0316	Progression/Metastasis	Yes
Clca4c-ps	−2.1	0.0257	NKR	
Plek2	−2.0	0.0000	Progression/Metastasis	Yes
Cldn4	−2.0	0.0000	Progression/Metastasis	Yes
Rpl31-ps14	−2.0	0.0457	Progression/Metastasis	
Fabp2	−2.0	0.0358	Protection	
Rbm11	−2.0	0.0445	Progression/Metastasis	Yes
Plat	−1.9	0.0095	Progression/Metastasis	Yes
Myrf	−1.9	0.0477	NKR	Yes
Cpn1	−1.8	0.0143	Progression/Metastasis	Yes
Smpd5	−1.8	0.0342	NKR	
Sema7a	−1.8	0.0032	Progression/Metastasis	Yes
Ndufb4b	−1.8	0.0009	Warburg	Yes
Tpbg	−1.7	0.0398	NKR	
Fam162a	−1.7	0.0016	NKR	
Atf3	−1.7	0.0000	NKR	Yes
Ttc39c	−1.6	0.0012	NKR	
Creb3l3	−1.6	0.0014	NKR	Yes
Fyb2	−1.6	0.0086	NKR	
H2bc24	−1.6	0.0353	NKR	
Ly6m	−1.6	0.0006	NKR	
Akr1b7	−1.6	0.0195	NKR	
Tnfrsf12a	−1.6	0.0003	Progression/Metastasis	
Ptp4a1	−1.5	0.0421	Progression/Metastasis	Yes
Smim3	−1.5	0.0005	NKR	
Hbegf	−1.5	0.0002	NKR	
Dusp10	−1.5	0.0027	Progression/Metastasis	Yes
Cda	−1.5	0.0015	NKR	
Rap2b	−1.4	0.0021	Progression/Metastasis	Yes
Dynlt1a	−1.4	0.0290	Progression/Metastasis	
Rpsa-ps1	−1.4	0.0343	Progression/Metastasis	
Ndufs5-ps	−1.4	0.0131	NKR	
Maff	−1.3	0.0010	Protection	Yes
Usp35	−1.3	0.0327	Progression/Metastasis	
Zfand4	−1.3	0.0455	NKR	
Ccdc141	−1.3	0.0389	NKR	
Duoxa2	−1.3	0.0019	NKR	
Capg	−1.3	0.0012	Progression/Metastasis	
Pla2g12a	−1.3	0.0032	Protection	Yes
Epha2	−1.2	0.0001	Progression/Metastasis	Yes
Nme4	−1.2	0.0208	NKR	
Trdc	−1.2	0.0408	NKR	
Arl14	−1.2	0.0062	NKR	
Ifrd1	−1.2	0.0076	Progression/Metastasis	Yes
S100a11	−1.2	0.0022	Progression/Metastasis	Yes
Gprc5a	−1.2	0.0017	Progression/Metastasis	Yes
Rhod	−1.2	0.0152	NKR	Yes
Rbp2	−1.2	0.0291	NKR	Yes
Hint3	−1.1	0.0020	NKR	Yes
Aldh3b2	−1.1	0.0013	Progression/Metastasis	
Sfn	−1.1	0.0001	NKR	Yes
Higd1a	−1.1	0.0273	Protection	Yes
Trim15	−1.1	0.0385	Progression/Metastasis	
Plaur	−1.1	0.0251	Progression/Metastasis	Yes
Avpi1	−1.0	0.0116	Progression/Metastasis	Yes
Pet100	−1.0	0.0144	NKR	Yes

* 63 of 151 UP/DOWN DEGs [long non-coding RNAs (lncRNAs), pseudogenes, or newly annotated and incompletely characterized DEGs] were excluded from these analyses. ** Abbreviations: NKR: No Known Relationship; ChIP: Chromatin Immunoprecipitation. *** Promoter engagement data from ENCODE and ChEA obtained from the Harmonizome website (https://maayanlab.cloud/Harmonizome/) built and maintained by the Ma’ayan Laboratory [12,13]-access date, 1 January 2020 denotes promoter occupation by STAT3 from ChIP studies conducted in multiple cell/tissues systems.

**Table 2:** DEGs (well-characterized) that were decreased by DSS and increased by TTI-101 (238 of 287 total UP/DOWN DEGs *): roles in CRC and promoter binding by STAT3.

Gene Symbol	TTI-101 vs. Veh (Log2FC)	*p*-Value	Role in CRC	STAT3 (ChIP) **^,^***
Slitrk3	7.6	0.0000	Progression/Metastasis	Yes
Bambi-ps1	7.0	0.0006	NKR	
Dlx2	7.0	0.0003	Progression/Metastasis	Yes
Nat1	7.0	0.0000	Protection	Yes
Gp6	6.9	0.0013	NKR	
Npy6r	6.9	0.0062	Progression/Metastasis	Yes
Xirp1	6.7	0.0015	NKR	Yes
Spata3	6.7	0.0017	NKR	Yes
Ighv5-6	6.4	0.0028	NKR	
Elovl2	6.4	0.0008	Progression/Metastasis	
Myoc	6.3	0.0047	NKR	
B3gat1	6.1	0.0239	Progression/Metastasis	Yes
Igkv5-39	6.1	0.0005	NKR	
Igkv4-55	5.9	0.0236	NKR	
Gdf7	5.9	0.0255	Progression/Metastasis	
Mzb1	5.8	0.0284	Protection	Yes
Jhy	5.8	0.0040	NKR	
Lrrc24	5.7	0.0169	Progression/Metastasis	
Chrna2	5.7	0.0002	NKR	
Syt10	5.7	0.0352	NKR	Yes
Gucy2g	5.7	0.0155	NKR	
Zfp982	5.4	0.0198	NKR	
Smok4a	5.0	0.0207	NKR	
Pak5	5.0	0.0382	Progression/Metastasis	
Clnk	4.9	0.0251	NKR	Yes
Adh7	4.8	0.0000	NKR	Yes
Eya1	4.7	0.0049	Progression/Metastasis	Yes
Ces2h	4.6	0.0265	NKR	
Gpr61	4.5	0.0015	NKR	
Igkv4-53	4.4	0.0076	NKR	
Hacl1	4.3	0.0000	NKR	Yes
7SK	4.3	0.0232	Protection	
Igkv19-93	4.2	0.0103	NKR	
Cers1	4.1	0.0029	Protection	
Xlr	4.0	0.0193	NKR	
Grid2ip	4.0	0.0017	Progression/Metastasis	
Lrrc9	4.0	0.0374	NKR	Yes
Klb	3.9	0.0005	Protection	
Maneal	3.8	0.0009	NKR	
Itih2	3.8	0.0208	Progression/Metastasis	
Igkv5-48	3.8	0.0086	NKR	
Htr7	3.7	0.0123	Protection	Yes
Crisp3	3.6	0.0138	NKR	Yes
Rims4	3.5	0.0008	NKR	
Igkv6-17	3.4	0.0449	NKR	
Soga3	3.3	0.0002	NKR	
Efcab9	3.1	0.0444	NKR	Yes
Chrm4	2.9	0.0441	Progression/Metastasis	Yes
Ighv1-26	2.9	0.0394	NKR	
Sfrp4	2.9	0.0001	Progression/Metastasis	
Ighg1	2.8	0.0081	Progression/Metastasis	
Glns-ps1	2.7	0.0166	NKR	
Cyp2f2	2.7	0.0020	NKR	
Hmx3	2.6	0.0223	Progression/Metastasis	
Cfap77	2.5	0.0402	NKR	
Aldh1a2	2.5	0.0097	Progression/Metastasis	
Dnah5	2.5	0.0035	NKR	
Spin4	2.4	0.0396	NKR	Yes
Ust	2.4	0.0009	NKR	Yes
Cckar	2.4	0.0242	Progression/Metastasis	
Adamts19	2.4	0.0033	Progression/Metastasis	Yes
Gfra3	2.3	0.0067	NKR	
Kcnd3	2.3	0.0000	NKR	Yes
H2-M2	2.3	0.0467	NKR	
Lrrn3	2.2	0.0027	NKR	Yes
Ptpn5	2.2	0.0227	Progression/Metastasis	Yes
Adcyap1r1	2.2	0.0001	NKR	
Neu3	2.1	0.0157	Progression/Metastasis	Yes
Grem2	2.1	0.0008	Protection	Yes
Kif26a	2.1	0.0102	Protection	Yes
Add2	2.1	0.0021	Protection	Yes
Npy1r	2.1	0.0078	Protection	Yes
Rasd2	2.0	0.0000	NKR	
Ptprn2	2.0	0.0049	Progression/Metastasis	Yes
Chrm2	1.9	0.0008	Progression/Metastasis	Yes
Nell2	1.9	0.0016	Progression/Metastasis	Yes
Colgalt2	1.9	0.0202	NKR	
Adh1	1.9	0.0160	Protection	
Myocd	1.8	0.0227	Progression/Metastasis	
Esrrg	1.8	0.0063	Protection	Yes
Diras2	1.8	0.0149	Protection	Yes
Sema3d	1.8	0.0040	Protection	Yes
Slc6a4	1.8	0.0128	Progression/Metastasis	
Hmcn1	1.8	0.0013	NKR	Yes
Efcc1	1.8	0.0147	NKR	Yes
Hhip	1.8	0.0014	NKR	Yes
Dubr	1.8	0.0000	NKR	
Xkr5	1.7	0.0032	NKR	
Col6a5	1.7	0.0061	NKR	Yes
Heyl	1.7	0.0403	Protection	
Kcnb1	1.7	0.0184	Protection	Yes
Astn1	1.7	0.0152	NKR	
BC034090	1.7	0.0025	NKR	
Rgs4	1.7	0.0003	NKR	Yes
Piwil4	1.7	0.0235	Progression/Metastasis	Yes
Abo	1.7	0.0000	Protection	Yes
Nxpe2	1.7	0.0027	NKR	Yes
Colec10	1.6	0.0075	NKR	Yes
Adgra2	1.6	0.0000	NKR	
Wasf3	1.6	0.0021	Progression/Metastasis	Yes
Col8a1	1.6	0.0412	Protection	Yes
P3h2	1.6	0.0164	Progression/Metastasis	Yes
Synm	1.6	0.0019	NKR	
Thbs4	1.6	0.0484	Protection	
Pld5	1.6	0.0466	NKR	Yes
Chrdl1	1.6	0.0074	Progression/Metastasis	
Slc15a1	1.6	0.0303	NKR	Yes
Syne3	1.6	0.0097	NKR	
Flt4	1.5	0.0001	NKR	
Gli3	1.5	0.0011	Protection	Yes
Lyve1	1.5	0.0002	NKR	
Pamr1	1.5	0.0010	Progression/Metastasis	Yes
Zfp791	1.5	0.0134	NKR	
Zfp128	1.5	0.0499	NKR	
Cadm3	1.5	0.0018	NKR	Yes
Adcy5	1.5	0.0000	NKR	
Scube2	1.5	0.0020	Progression/Metastasis	
Col23a1	1.5	0.0026	Progression/Metastasis	Yes
Ppp1r3c	1.5	0.0251	Protection	Yes
Rspo3	1.4	0.0335	Protection	
She	1.4	0.0022	NKR	
Synpo2	1.4	0.0008	Protection	Yes
Sgtb	1.4	0.0368	NKR	Yes
Lbp	1.4	0.0398	NKR	Yes
Ano1	1.4	0.0255	Protection	Yes
Stum	1.4	0.0010	NKR	
Hpcal4	1.4	0.0044	NKR	
Rragd	1.4	0.0003	NKR	
Slc6a17	1.4	0.0011	NKR	Yes
Tns1	1.4	0.0000	NKR	
Pianp	1.4	0.0125	NKR	
Peli2	1.4	0.0014	NKR	Yes
Id4	1.4	0.0013	Progression/Metastasis	Yes
Abi3bp	1.4	0.0047	NKR	Yes
Nipal4	1.3	0.0216	NKR	Yes
Prickle2	1.3	0.0066	NKR	Yes
Brdt	1.3	0.0074	NKR	
Kcnf1	1.3	0.0019	NKR	
Arhgap28	1.3	0.0102	NKR	
Galnt15	1.3	0.0016	NKR	
Exph5	1.3	0.0133	NKR	
Ace	1.3	0.0018	Progression/Metastasis	Yes
Syn2	1.3	0.0100	NKR	Yes
Tgfbr3	1.3	0.0000	NKR	
Negr1	1.3	0.0357	Protection	Yes
Prkcb	1.3	0.0099	Progression/Metastasis	Yes
Slc5a6	1.3	0.0141	NKR	
Slco3a1	1.3	0.0038	NKR	Yes
Serpinf1	1.3	0.0023	NKR	
St3gal6	1.3	0.0391	Progression/Metastasis	Yes
Fibin	1.3	0.0381	NKR	Yes
Lmod1	1.3	0.0020	NKR	Yes
Nynrin	1.3	0.0001	NKR	
Fgd5	1.3	0.0080	Progression/Metastasis	
Bnc2	1.3	0.0253	NKR	
Sacs	1.3	0.0093	NKR	
Evc	1.2	0.0020	NKR	Yes
Dse	1.2	0.0115	NKR	Yes
Gli2	1.2	0.0107	Protection	
Fgfr1	1.2	0.0007	Protection	Yes
Clec3b	1.2	0.0220	NKR	Yes
Rapgef5	1.2	0.0031	NKR	Yes
Mmrn1	1.2	0.0052	Protection	
Pdzd2	1.2	0.0044	NKR	Yes
Bves	1.2	0.0126	Progression/Metastasis	
Slit3	1.2	0.0070	Progression/Metastasis	Yes
Lum	1.2	0.0117	NKR	Yes
Cbfa2t3	1.2	0.0026	NKR	Yes
Itga8	1.2	0.0184	NKR	
Sun2	1.2	0.0051	Protection	Yes
Ric3	1.2	0.0147	NKR	
Ccl28	1.2	0.0102	Progression/Metastasis	Yes
Reln	1.2	0.0038	NKR	Yes
Ecm2	1.2	0.0058	NKR	Yes
St6galnac4	1.2	0.0219	NKR	Yes
Flnc	1.2	0.0179	NKR	
Apbb1	1.2	0.0131	NKR	
Mr1	1.2	0.0179	NKR	Yes
Alox5	1.2	0.0077	NKR	
Fzd2	1.2	0.0000	NKR	Yes
Plcb1	1.2	0.0003	NKR	Yes
Itgb3	1.2	0.0238	NKR	Yes
Daam2	1.2	0.0056	NKR	
Tspan33	1.2	0.0007	NKR	Yes
Insm1	1.2	0.0171	NKR	
Vps13c	1.1	0.0249	NKR	
Rassf2	1.1	0.0005	NKR	
Gask1b	1.1	0.0311	NKR	
Trpc1	1.1	0.0067	Protection	
Zfp526	1.1	0.0307	NKR	
Gdf10	1.1	0.0283	Protection	Yes
Egf	1.1	0.0185	Progression/Metastasis	Yes
Itih5	1.1	0.0325	NKR	
Nrp2	1.1	0.0000	Protection	Yes
C79130	1.1	0.0280	NKR	
Kit	1.1	0.0031	NKR	Yes
Setbp1	1.1	0.0315	Progression/Metastasis	Yes
B3gnt9	1.1	0.0182	NKR	
Epha7	1.1	0.0105	NKR	Yes
Emid1	1.1	0.0121	NKR	Yes
Sema5a	1.1	0.0169	NKR	Yes
Prkg1	1.1	0.0457	NKR	Yes
Kcna2	1.1	0.0370	NKR	Yes
Cntln	1.1	0.0147	NKR	Yes
Dchs1	1.1	0.0279	NKR	Yes
Ppp1r12b	1.1	0.0015	NKR	Yes
Ldb2	1.1	0.0077	NKR	Yes
Pid1	1.1	0.0184	NKR	Yes
Sv2c	1.1	0.0270	NKR	Yes
Htr4	1.1	0.0128	NKR	
Hip1	1.1	0.0014	NKR	
Dcbld2	1.1	0.0262	Protection	Yes
Rnf144b	1.1	0.0001	NKR	Yes
Slc25a12	1.1	0.0004	NKR	Yes
Zfhx4	1.1	0.0398	NKR	
Pgm5	1.1	0.0075	Progression/Metastasis	
Acss2	1.0	0.0038	NKR	Yes
Ogn	1.0	0.0039	Progression/Metastasis	
Nr5a2	1.0	0.0187	NKR	Yes
Aqp1	1.0	0.0271	NKR	Yes
Akap12	1.0	0.0014	Protection	Yes
Casq2	1.0	0.0424	NKR	
Lrrc3	1.0	0.0198	NKR	
Slc9a3r2	1.0	0.0031	Progression/Metastasis	
Arhgap31	1.0	0.0054	NKR	Yes
Zeb1	1.0	0.0029	Progression/Metastasis	
Pfkm	1.0	0.0005	NKR	Yes
Tmem231	1.0	0.0252	NKR	
Myo5a	1.0	0.0033	NKR	
Itpkb	1.0	0.0003	NKR	Yes
Cdon	1.0	0.0007	NKR	Yes
Gucy1a1	1.0	0.0125	NKR	
Acsm3	1.0	0.0384	NKR	Yes
Filip1	1.0	0.0013	NKR	Yes
Flna	1.0	0.0012	Protection	Yes
Kcnma1	1.0	0.0033	Progression/Metastasis	Yes
Chst4	1.0	0.0248	NKR	
Irag1	1.0	0.0038	NKR	

* 49 of 287 DOWN/UP DEGs that were long non-coding RNAs(lncRNA), pseudogenes, or newly annotated poorly described DEGs were excluded, leaving 238 DEGs for these analyses. ** Abbreviations: NKR: No Known Relationship, ChIP: Chromatin Immunoprecipitation. *** Promoter engagement data from ENCODE and ChEA obtained from the Harmonizome website (https://maayanlab.cloud/Harmonizome/) built and maintained by the Ma’ayan Laboratory [12,13], denotes promoter occupation by STAT3 from ChIP studies conducted in multiple cell/tissues systems.

## Data Availability

The array data will be uploaded to the NCBI GEO database.

## Abbreviations

The following abbreviations are used in this manuscript:

CRC	Colorectal cancer
IBD	Inflammatory bowel disease
UC	Ulcerative colitis
STAT3	Signal transducer and activator transcription 3
DSS	Dextran sodium sulfate
AOM	Azoxymethane
PCoA	Microbiome principal coordinates analysis
IPA	Ingenuity pathway analyses
DEGs	Differentially expressed genes
